# Peritumoral T2/FLAIR hyperintense MRI findings of meningiomas are not necessarily edema and may persist permanently: a systematic review

**DOI:** 10.1007/s10143-023-02094-1

**Published:** 2023-08-05

**Authors:** Joonas Laajava, Miikka Korja

**Affiliations:** Department of Neurosurgery, University of Helsinki and Helsinki University Hospital, P.O. Box 320, Haartmaninkatu 4, FI-00290 Helsinki, Finland

## Abstract

The current knowledge regarding the prevalence and persistence of edematous changes postmeningioma surgery is limited. Our hypothesis was that peritumoral edema is frequently irreversible gliosis, potentially influencing long-term postoperative epilepsy. We conducted a systematic literature search in PubMed, Cochrane Library, and Scopus databases. We included studies with adult patients undergoing first supratentorial meningioma surgery, which reported pre- and postoperative peritumoral brain edema (T2WI and FLAIR hyperintensity on MRI). Risk of bias was assessed based on detailed reporting of five domains: (1) meningioma characteristics, (2) extent of resection, (3) postoperative radiation therapy, (4) neurological outcome, and (5) used MRI sequence. Our loose search strategy yielded 1714 articles, of which 164 were reviewed and seven met inclusion criteria. Persistent edema rates ranged from 39% to 83% with final follow-up occurring between 0, 14, and 157 months. Among patient cohorts exhibiting persistent edema, a smaller portion achieved seizure resolution compared to a cohort without persistent edema. Relatively reliable assessment of persistent T2/FLAIR hyperintensity changes can be made earliest at one year following surgery. All studies were classified as low quality of evidence, and therefore, quantitative analyses were not conducted. Persistent T2/FLAIR hyperintensity changes are frequently observed in MRI imaging following meningioma surgery. The term “edema,” which is reversible, does not fully capture pre- and postoperative T2WI and FLAIR hyperintensity changes. Future studies focusing on peritumoral meningioma-related edema, its etiology, its persistence, and its impact on postoperative epilepsy are needed.

## Introduction

Intracranial meningiomas (IMs) are the most frequently diagnosed brain tumors representing over a third of primary brain tumors [1]. The incidence of IM is approximately 8 per 100,000 population, incidence increases with age and displays a woman-to-man ratio of approximately two-to-one [1]. Meningiomas can originate at any location on the intracranial or spinal dural surface [2]. Typically, IMs grow slowly and cause focal symptoms [2]. Many are discovered incidentally on brain imaging [2]. The diagnosis and monitoring of IM growth are nowadays accomplished through the utilization of magnetic resonance imaging (MRI) [2].

Peritumoral brain edema (PTBE) is a common feature observed in IMs on MRI imaging [2, 3]. Peritumoral edema is typically defined as hyperintensity on T2WI or FLAIR sequences [2, 4]. PTBE has been demonstrated to be more frequent in brain-invasive and secretory IMs [5–7]. Moreover, in convexity and parasagittal meningiomas, PTBE has been shown to have a positive correlation with tumor size [8]. PTBE is also more common in older patients [9]. PTBE has been linked with longer hospital stays, a higher requirement for postoperative medical assistance, and higher short- and long-term mortality rates [10]. Preoperative PTBE has also been linked with higher recurrence rates [10–12]. Patients with preoperative PTBE have been found to have significantly higher rates of pre- and postoperative seizures [13–16].

Following surgical excision of IM, a disappearance in PTBE is sometimes observed in following months. The time course of this disappearance has been shown to vary between patients [17]. Earlier studies using a computer tomography (CT) follow-up have reported that edema persists in a significant number of patients. Stevens et al. demonstrated that 13% of patients experienced persistent postoperative PTBE for at least three months [18]. In a smaller study conducted by Vignes et al., up to 30% of patients had persistent edema at 1-year follow-up [19].

The MRI representations of persistent edema and gliosis on FLAIR and T2 sequences share striking similarities [20], presenting a challenge when trying to differentiate between these conditions. Persistent edema has previously been suggested to represent a combination of vasogenic brain edema and cerebral gliosis [21]. Hence, throughout this article, we will refer to these changes as only T2/FLAIR hyperintense changes. To aid in distinguishing reactive gliosis from vasogenic edema, T2 diffusion-weighted MR imaging alongside apparent diffusion coefficient maps (ADC) can be used [20]. However, the sole definitive method to differentiate between edema and gliosis is through biopsy. Obtaining such samples is fraught with ethical complexities and challenging to justify.

The objective of this study was to look at the incidence of persistent “edematous” T2 or FLAIR-signal changes following surgery of IMs. Additionally, we aimed to assess whether persistent MRI changes associate with postoperative epilepsy and whether specific histological characteristics of IMs associate with the occurrence of persistent signal changes.

## Materials and methods

Our systematic review was guided by the Preferred Reporting Items for Systematic Review and Meta-Analyses (PRISMA) checklist [22]. This review is registered in the Prospero [23].

### Study selection

We based our study question on the four-step PICO (patient, intervention, comparison, outcome) principle [24]. We conducted our literature search using PubMed, Scopus, and Cochrane library. We first carried out the searches on December 11, 2022, and re-ran them on April 24, 2023. To include studies in this review, we set the following criteria: (1) the study participants had to be adults (> 18 years of age), (2) the participants must have undergone their first meningioma surgery, (3) the surgery must have been performed for supratentorial meningiomas, (4) the studies must report data on pre- and postoperative-T2/FLAIR hyperintensity, and (5) postoperative follow-up had to be performed with MRI imaging instead of CT. We excluded book chapters, case reports, case series (*n* < 5), letters, reviews, commentaries, and animal studies from the review. We did not place any restrictions on publication year or language.

### Data extraction

We conducted data extraction on common patient demographics, such as age and gender, as well as on preoperative tumor characteristics including size, location, T2/FLAIR hyperintensity, and histology. Additionally, we collected information on the extent of resection, duration of MRI follow-up, recurrence rates, and the presence or disappearance of postoperative T2/FLAIR hyperintense changes. We also aimed to collect data on neurological outcome with special emphasis on seizures. For the final analyses, we tried to include only patients with confirmed gross total resection (GTR).

### Quality assessment

We performed the quality assessment of each study by using a domain-based evaluation approach as suggested by Cochrane Collaboration Handbook [25]. We used five different domains for this evaluation: (1) preoperative IM characteristics that included location, size, histology, and presence of T2/FLAIR hyperintensity; (2) extent of resection, which meant that studies were required to report the extent of surgical resection; (3) postoperative radiotherapy (reported or not), in order to exclude cases with postoperative radiation-induced T2/FLAIR hyperintensity [26]; (4) postoperative neurological outcome, particularly focusing on seizures; and (5) used MRI sequence, as the imaging parameters impact the detection of T2/FLAIR hyperintensity.

Based on these domains, we classified studies as low, unknown, or high risk of bias. To be classified as a low risk of bias study (i.e., a high-quality study), we required that it fulfilled and reported each domain. If one or more domains were missing, the study was considered to have a high risk of bias, resulting in its classification to the low-quality category. Studies were also classified as low quality if the reported information contained contradictory or conflicting methodologies. If the domains were reported only partially, this led to the unknown bias category in the domain assessment.

## Results

### Study selection

**Fig. 1:**
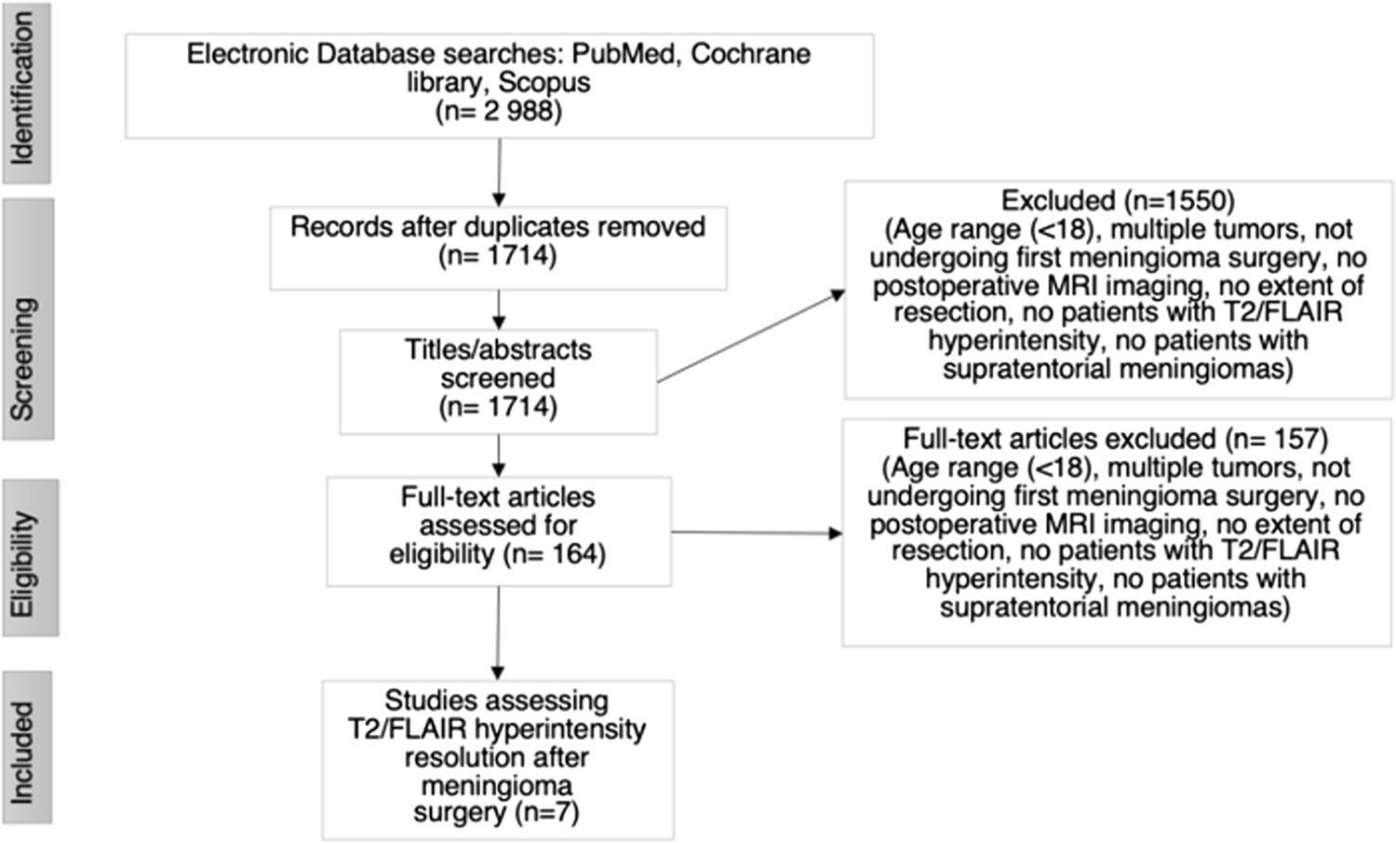
Study selection process

The study selection protocol is illustrated in Fig. 1. After removing duplicates, we screened a total of 1714 article abstracts for eligibility. Following the screening process, we read 164 articles and identified seven articles that fulfilled our inclusion criteria [27–32]. Of the identified seven articles, four were retrospective case series (years 1998–2023) [29–32], one was a retrospective cohort study (year 2018) [33], one was a prospective study (year 2019) [28], and one was a retrospective matched pairwise analysis (year 2015) [27]. All seven studies were conducted between 1990 and 2020 Geographically, four studies were conducted in the USA [29, 31–33] and the remaining three in Canada [27], Germany [31], and Bosnia and Herzegovina [28]. The study conducted in Germany was written in German. Overall, the studies comprised a total of 221 patients, and the number of participants varied between 14 and 75. Of the participants, 82 were men (37,1%). A more detailed description of the study characteristics is depicted in Table 1.

**Table 1:** General characteristics of selected studies

First author, year and reference	Country	Study period	Patients	Age, mean (range)	Men (%)	Study design
Almeida, 2015 [27]	Canada	2003–2012	20	51,4 (N/A)	25	Retrospective pairwise analysis
Bečulić, 2019 [28]	Bosnia and Herzegovina	2010–2015	41	63,0 (N/A)	41,5	Prospective study
Bander, 2018 [33]^a.^	USA	2000–2015	32	55,0 (28–80)	37,5	Retrospective cohort study
Prevedello, 2015 [29]	USA	1998–2008	18	54,4 (35–75)	22,2	Retrospective case study
Bitzer, 1999 [30]	Germany	N/A	14	49,1 (32–64)	21,4	Retrospective case series
Dolinskas, 1998 [31]	USA	1990–1993	21	56 (32–74)	47,6	Retrospective case series
Champagne, 2023 [32]	USA	2003–2020	75	60,6 (N/A)	41	Retrospective case series

*N/A* not available

^a.^Published online 2017

### Meningioma characteristics

Only two of the selected studies [30, 32] provided detailed data about IM characteristics, such as size, histology, location, and T2/FLAIR hyperintensity volume. Three studies did not report histology [27, 29, 33]. One study [28] failed to report the meningioma location. One study did not report the meningioma size [31], and two studies failed to report preoperative T2/FLAIR hyperintensity volume [31, 32]. These two studies did, however, report the number of patients with preoperative T2/FLAIR hyperintensity. The mean tumor size varied between 5,04 cm^3^ and 40,47 cm^3^. The largest reported tumor volume was 80,8 cm^3^, and the smallest 0,58 cm^3^. The mean T2/FLAIR hyperintensity volume varied between 2,45 cm^3^ and 52,5 cm^3^. The largest T2/FLAIR hyperintensity volume was 127,5 cm^3^. For more detailed meningioma characteristics, please see Table 2.

**Table 2:** Preoperative meningioma characteristics

First author, year and reference	Mean tumor size (range) (cm^3^)	Preoperative tumor location (patients)	Mean T2/FLAIR hyperintense volume (cm^3^)	Histology (patients)
Almeida, 2015 [27]	35,95 (N/A)	Olfactory groove (20)	29,05 (N/A)	N/A
Bečulić, 2019 [28]	27,81 (19,04–36,58)	N/A	4,87 (3,58–6,15)	WHO Grade I (34) WHO Grade II (6) WHO Grade III (1)
Bander, 2018 [33]^a.^	5,33 (0,58–13,64)	Tuberculum sellae (N/A) Planum sphenoidale (N/A)	3,24 (0,00–23,65)	N/A
Prevedello, 2015 [29]	20,92 (2,8–75,7)	Olfactory groove (18)	30,4 (0–127,5)	N/A
Bitzer, 1999 [30]	40,47 (11,8–80,8)	Sphenoid wing (2) Falx (3) Skull (1) Temporobasal (1) Frontobasal (1) “Sinuskante rechts” (1)	52,5 (2,2–122,7)	WHO Grade I (9)
Dolinskas, 1998 [31]	N/A	Frontal (4) Sphenoid wing (3) Petrous (1) Cerebellopontine (1) Tuberculum sellae (1) Suprasella (1) Middle cranial fossa (1) Frontotemporal (1) Foramen magnum (1) Parietal (1) Anteriol clinoid (1) Frontoparietal (1) Olfactory groove (1) Parietooccipital (1) Planum sphenoidale (1)	N/A^c.^	WHO Grade I (17) WHO Grade II (3) WHO Grade III (1)
Champagne, 2023 [32]	43,3 (N/A)	Olfactory groove (75)	N/A^c.^	WHO Grade I (72) WHO Grade II (3)

*N/A* not available

^a.^Published online 2017

^b.^Patient details removed for five patients (two infratentorial meningiomas and three multiple meningiomas)

^c.^Preoperative T2/FLAIR hyperintensity reported as number of patients

### Meningioma locations

Of the 221 meningiomas included in the analysis, the location was reported for 175. The most common (114 meningiomas, 65%) location was the olfactory groove. Thirty-four meningiomas (19%) are located in the tuberculum sellae/planum sphenoidale. The rest of the locations were the sphenoid wing (five meningiomas, 3%), frontal convexity (four meningiomas, 2%), and falx (three meningiomas, 2%). We classified the location for 15 (9%) meningiomas as “other,” since each 15 had varying locations.

### Histology

Meningioma histology was reported for 146 (66%) tumors. Of these, 132 meningiomas (91%) were WHO Grade I, 12 (8%) WHO Grade II, and two (1%) WHO Grade III.

### *Extent of resection*

All studies reported the extent of resection (EOR). EOR was reported as either gross- (GTR), near- (NTR), or subtotal resection (STR) by two studies [29, 33]. Three studies included Simpson grading in their assessment of EOR [27, 28, 32]. In the study by Champagne et al. [32], 48 out of 75 patients were reported as having EOR as Simpsons grade I. We classified the remaining patients as NTR if EOR was > 90% and STR if EOR was < 90%. One study [30] reported any residual tumor as an exclusion criterion, indicating that all patients underwent GTR. One study [31] reported on whether the patients had any residual tumor. We classified patients with residual tumor as STR and those, without as GTR. Complete EOR details are presented in Table 3.

**Table 3:** Postoperative variables of interest

First author, year and reference	Extent of resection (number of patients)	Neurological outcome	MRI follow-up in months	Recurrences at last follow-up (%)
Almeida, 2015 [27]	GTR (16) STR (4)	1 stroke	4,4	2 (10%)
Bečulić, 2019 [28]	GTR (39) NTR (2)	N/A	3	N/A
Bander, 2018 [33]^a.^	GTR (24) NTR (7) STR (3)	4 seizures 2 SAH/stroke 1 weakness	N/A	4 (12,5%)
Prevedello, 2015 [29]	GTR (18)	N/A	5,8 (0,14–20)	N/A
Bitzer, 1999 [30]	GTR (9)	N/A	> 6	N/A
Dolinskas, 1998 [31]	GTR (14) STR (7)	N/A	1–2, 6, 12	4 (19%)
Champagne, 2023 [32]	GTR (48) NTR (12) STR (15)	11 neurological complications	45,8	11 (15%)

*N/A* not available, *SAH* subarachnoid hemorrhage

Extent of resection: GTR (gross-total resection) = 100% tumor removal, NTR (near-total resection) > 90% tumor removal, STR (subtotal resection) < 90% tumor removal

^a.^Published online 2017

For the purposes of analysis, we classified Simpsons Grade I and II excisions as GTR, Grade III as NTR, and Grade IV as STR [34]. In summary, out of the 221 patients, 166 underwent GTR, 21 NTR, and 29 STR [27–32]. The remaining five patients were excluded since two of them had infratentorial meningiomas and three multiple meningiomas (Table 2) [30].

### Follow-up MRI imaging and postoperative T2/FLAIR hyperintensity

Except for one study [33], all reported the timing of MRI follow-up imaging. One study [30] reported that MRI-imaging took place at least six months after operation, without providing exact timing. Across the studies, there was variance in imaging frequency and interval. MRI follow-up times varied between 0,14 and 157 months; the short 0,14-month follow-up was due to a patient mortality in the study [29]. Only three studies stated clearly that patients did not receive postoperative radiotherapy. These studies allowed us to exclude that radiation-induced hyperintensity accounted for postoperative MRI changes [27, 29, 32].

**Fig. 2:**
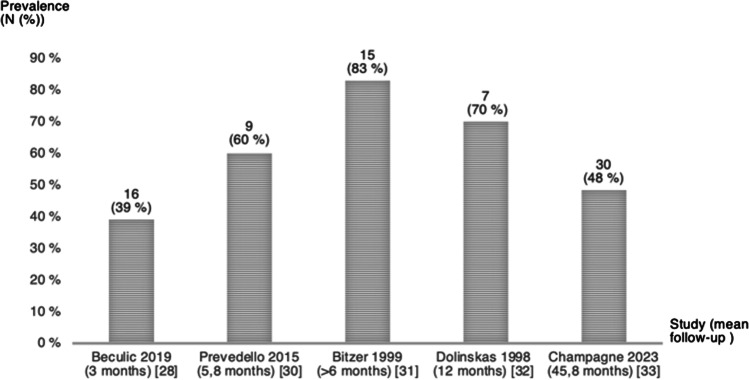
Prevalence of postoperative T2/FLAIR hyperintensity changes in patients with initial hyperintensity

All studies reported on the presence of postoperative T2/FLAIR hyperintensity. However, there were differences in reporting. Three of the studies [29, 32, 33] assessed hyperintensity on FLAIR sequence, while three studies [27, 28, 30] assessed hyperintensity on T2WI. One study only talked about “edema” without mentioning on what sequence hyperintensity was assessed [31]. Two studies [27, 33] failed to provide the number of patients with postoperative T2/FLAIR hyperintensity. In these two studies, the presence of postoperative T2/FLAIR hyperintensity was reported as a mean number within different patient groups based on surgical approach used for resection. The remaining five studies [28–32] reported the number of patients with postoperative T2/FLAIR hyperintensity. In these five studies [28–32], the presence of postoperative T2/FLAIR hyperintensity in patients with initial hyperintense changes varied between 39% and 83% at the last follow-up. The remaining patients had hyperintensity resolve. Prevalence of postoperative T2/FLAIR hyperintensity and mean follow-up time in months for each study [28–32] is depicted in Fig. 2. One study [29] reported MRI follow-up times for each patient separately.

Five studies reported on mean T2/FLAIR hyperintense volume [27–30]. In four of these studies [27–30], there was a mean reduction in T2/FLAIR hyperintense volume in the follow-up period. The follow-up period varied from 0,14 months to 20 months in these four studies. Additionally, the study by Prevedello et al. [29], which reported data of individual patients, reported FLAIR signal reduction in all 13 patients with preoperative FLAIR hyperintensity changes. In immediate postoperative imaging, Prevedello et al. did report one case of increased FLAIR hyperintensity. This presumably iatrogenic finding reduced in volume in follow-up imaging. The study by Bander et al. [33] reported a mean increase in FLAIR hyperintensity volume of 4,15 cm^3^ among 15 patients. Unfortunately, the timing of the MRI follow-up in months was not reported, and individual patient was data not available.

In our review, there were reports of FLAIR hyperintensity persisting beyond a year. Specifically, Prevedello et al. [29] documented a case in which there was only a 92% reduction in FLAIR hyperintensity, compared to the preoperative images, at a 20-month MRI follow-up. Most notably, Champagne et al. [32] reported the longest instances of persistent FLAIR hyperintensity. In their study, which included the most extended follow-up periods, they observed that in a cohort of 50 patients who underwent surgery before 2014, over 50% exhibited enduring FLAIR hyperintensity in MRI over 8 to 9 years postoperatively.

### Seizures and postoperative neurological outcome

Three studies reported on postoperative neurological outcome [27, 32, 33]. These studies included 75 patients, of which 20 patients experienced postoperative neurological complications. Bander et al. [33] reported four cases of new postoperative seizures in patients. None of these patients had prior history of seizures. Champagne et al. [32] reported that of 15 patients suffering from preoperative seizures only six became seizure free in the mean follow-up time of 45,8 month. In either case no further comment was made on how many of these patients had preoperative T2/FLAIR hyperintensity. Further details regarding neurological outcome can be found in Table 3.

### Quality assessments

None of the included studies met our criteria for high quality. Several major shortcomings and potential sources of biases were identified across the studies. Firstly, limited reporting of IM characteristics was observed in five studies [27–29, 31]. Secondly, four studies [28, 30, 31, 33] failed to adequately report postoperative radiotherapy details. Thirdly, four studies [28–31] did not report neurological outcome. Lastly, one study [31] did not report the MRI sequence used for assessing hyperintensity.

## Discussion

### Main findings

For our surprise, we found only seven studies that have investigated the evolution of peritumoral T2/FLAIR hyperintense changes following meningioma surgery. All these studies [27–32] reported the presence of T2/FLAIR hyperintense changes in some patients at the last follow-up MRI, which varied between 0,14 and 157 months. These findings suggest that preoperative T2/FLAIR hyperintensity does not necessarily represent a true reversible edematous hyperintense change but gliosis. If true, better understanding of the pathophysiological phenomena behind the peritumoral T2/FLAIR MR changes could improve the surgical strategy and outcome prediction in meningioma surgery. For instance, if permanent meningioma-related T2/FLAIR MR changes are an epileptic focus, it seems unlikely that preoperatively occurred seizures will come to an end without antiepileptic medication following meningioma resection. Similarly, if a patient suffers from motor weakness related to the permanent T2/FLAIR MR changes, it is conceivable that surgery will not resolve all motor problems. Unfortunately, none of the reviewed studies reported whether patients with complete postoperative T2/FLAIR hyperintense resolution had improved symptoms or better neurological outcome. Moreover, only two studies [32, 33] touched briefly upon postoperative seizures and T2/FLAIR MR changes. Therefore, future studies need to assess the association of permanent T2/FLAIR MR changes (i.e., possible gliosis) and neurological outcome, particularly with regard to epilepsy.

### Postoperative T2/FLAIR hyperintensity following surgery

The evolution of T2/FLAIR hyperintense changes following meningioma surgery is poorly studied. Two studies [27, 33] reported postoperative T2/FLAIR hyperintensity changes only as a mean number among study participants, but they did not provide insights into this phenomenon at the individual level. Interestingly, in the study by Bander et al. [33], study patients showed a mean increase in postoperative FLAIR hyperintense volume of 4,73 cm^3^. However, the study did not report the time point of postoperative MRI imaging, and it is likely that it took place in a very early phase. This increase in postoperative FLAIR hyperintense changes may be complication-related, as it is known that for example venous sacrifice during meningioma surgery can lead to an increased postoperative T2/FLAIR hyperintensity [35]. All other studies reported reduction or disappearance of T2/FLAIR hyperintensity following surgery.

Perhaps the most comprehensive data on the evolution of T2/FLAIR hyperintense changes come from two studies [29, 30], which included only patients who underwent GTR (no residual tumor contributing to T2/FLAIR hyperintense changes). Both studies also reported the postoperative treatment protocol in such a way that we were able to exclude the possibility that T2/FLAIR hyperintense changes were related to postoperative radiotherapy. Furthermore, these two studies described postoperative complications in detail, which allowed us to exclude T2/FLAIR hyperintense changes stemming from intraoperative venous sacrifice.

Prevedello et al. [29] reported that the quickest complete resolution of T2/FLAIR hyperintensity was observed in 5 months in a patient with a relatively small preoperative FLAIR hyperintensity volume of 6,6 cm^3^. Interestingly, another patient with a preoperative FLAIR hyperintense volume of 89,0 cm^3^ had a near complete (99% reduction to 0,7cm^3^) resolution in only 4 months [29]. On the contrary, in one patient, FLAIR hyperintensity changes were still present at 20 months following surgery [29]. Bitzer et al. [30] reported the highest prevalence of persistent T2/FLAIR hyperintensity changes, as out of 18 operated meningioma patients, 15 (83%) showed T2 hyperintensity changes at follow-up MRI at six months or later. In a detailed analysis of the location of postoperative T2 hyperintensity changes, seven patients (39%) showed changes within the same location than preoperative T2 hyperintensity [30]. In two patients (11%), there was only partial overlap, and in other two patients (11%), the location of hyperintensity did not correspond to the preoperative location and is therefore likely attributed to intraoperative vascular damage.

The remaining studies [28, 31, 32] did not report postoperative T2/FLAIR hyperintense changes by EOR. Bečulić et al. [28] conducted their follow-up imaging at a single time point of 3 months. Moreover, the number of patients with preoperative T2 hyperintensity changes was not reported. The study conducted by Champagne et al. [32] is noteworthy as all patients operated after 2014 showed complete resolution of FLAIR hyperintense changes within four years. Interestingly, some of the patients operated before 2014 appeared to have a late resolution of these changes even after eight to nine years [32]. It is challenging to speculate about the mechanisms explaining the late resolution. Of the reviewed studies, only Dolinskas et al. [31] conducted a baseline MRI immediately post-surgery and follow-up MRIs at three distinct time points. Interestingly, six patients with preoperative “edema” exhibited an initial decrease in “edema” volume within the first eight weeks (average 36 days) but part of the changes persisted in the follow-up MRIs and transformed according to the authors into an appearance consistent with gliosis. In summary T2/FLAIR hyperintense. changes may disappear completely in five months, but in some patients, it persists and may in fact represent iatrogenic gliosis.

### Seizures related to T2/FLAIR hyperintensity

With regard to T2/FLAIR hyperintensity-related seizures, the data is also scarce. Bander et al. [33] reported four patients with postoperative seizures, but the preoperative symptoms and postoperative T2/FLAIR hyperintense findings were not reported in detail. In the study by Champagne et al. [32], of the 15 patients who had preoperative seizures, six became seizure free during the postoperative follow-up. Of note, of the three patients with complete postoperative FLAIR hyperintense resolution, one still had postoperative seizures during the mean follow-up time of 45.8 months of the whole cohort. A few previous studies have suggested that preoperative T2/FLAIR hyperintensity is associated with a higher incidence of postoperative seizures [13–16]. Based on our review, the current literature does not show reliable evidence that would associate preoperative T2/FLAIR hyperintensity or postoperative T2/FLAIR hyperintense changes (possible gliosis) with epileptic seizures. Further research is needed to better understand the relationship between T2/FLAIR hyperintensity, preoperative symptoms and postoperative outcome.

## Limitations

While our review provides insights into the topic, it is important to acknowledge its limitations. First, as usual, there is a possibility that some relevant studies may have been missed. Second, due to the quality issue of included articles, we were only able to conduct qualitative analyses about the topic. Third, for our surprise, most (84%) of the reported cases located in only three specific locations: the olfactory groove, tuberculum sellae, and planum sphenoidale. Therefore, the reported findings may not apply to meningiomas occurring in other locations. Despite the shortcomings, our systematic review summarizes the existing evidence on the evolution of T2/FLAIR hyperintensity following surgery, highlighting the important areas for future research. As T2/FLAIR hyperintensity has surely a significant role in surgery and meningioma-related symptoms, hopefully our review increases interest on the topic.

## Conclusion

Persistent T2/FLAIR hyperintensity is frequently observed on MRI following meningioma surgery. Since T2/FLAIR hyperintensity changes may continue to resolve years after surgery, the assessment of persistent changes should not be made within the first year following surgery. In some instances, persistent T2/FLAIR hyperintensity changes may represent reactive or iatrogenic gliosis. Therefore, it is important to recognize that the term “edema” may not describe accurately these peritumoral imaging changes. In cases where T2/FLAIR hyperintensity changes persist over a year, it is perhaps reasonable to consider the finding as permanent gliosis, particularly in patients suffering from seizures. Further research with larger sample sizes and more detailed preoperative and postoperative data is needed to provide a more conclusive understanding of the potential impact of persistent T2/FLAIR hyperintensity on postoperative outcome.

## Data Availability

Not applicable.
